# Motor correlates of phantom limb pain

**DOI:** 10.1016/j.cortex.2017.07.015

**Published:** 2017-10

**Authors:** Sanne Kikkert, Melvin Mezue, David Henderson Slater, Heidi Johansen-Berg, Irene Tracey, Tamar R. Makin

**Affiliations:** a FMRIB Centre, Nuffield Department of Clinical Neurosciences, University of Oxford, Oxford, United Kingdom; b Donders Institute for Brain, Cognition and Behaviour, Radboud University Nijmegen, Nijmegen, The Netherlands; c Oxford Centre for Enablement, Nuffield Orthopaedic Centre, Oxford, United Kingdom; d Nuffield Division of Anaesthetics, University of Oxford, Oxford, United Kingdom; e Institute of Cognitive Neuroscience, University College London, London, United Kingdom

**Keywords:** Amputees, Body representation, Neuroimaging, Phantom limb pain, Plasticity

## Abstract

Following amputation, individuals ubiquitously report experiencing lingering sensations of their missing limb. While phantom sensations can be innocuous, they are often manifested as painful. Phantom limb pain (PLP) is notorious for being difficult to monitor and treat. A major challenge in PLP management is the difficulty in assessing PLP symptoms, given the physical absence of the affected body part. Here, we offer a means of quantifying chronic PLP by harnessing the known ability of amputees to voluntarily move their phantom limbs. Upper-limb amputees suffering from chronic PLP performed a simple finger-tapping task with their phantom hand. We confirm that amputees suffering from worse chronic PLP had worse motor control over their phantom hand. We further demonstrate that task performance was consistent over weeks and did not relate to transient PLP or non-painful phantom sensations. Finally, we explore the neural basis of these behavioural correlates of PLP. Using neuroimaging, we reveal that slower phantom hand movements were coupled with stronger activity in the primary sensorimotor phantom hand cortex, previously shown to associate with chronic PLP. By demonstrating a specific link between phantom hand motor control and chronic PLP, our findings open up new avenues for PLP management and improvement of existing PLP treatments.

## 1 Introduction

Following arm amputation individuals generally perceive vivid sensations of the amputated limb as if it is still present, with varying ability to voluntarily move this phantom hand. In up to 80% of arm amputees these phantom sensations are experienced as painful and can manifest as an intractable chronic neuropathic pain syndrome (Weeks, Anderson-Barnes, & Tsao, 2010). Phantom limb pain (PLP) often does not respond to conventional analgesic therapies and poses a significant medical problem (Knotkova, Cruciani, Tronnier, & Rasche, 2012).

A large number of studies have associated PLP with plastic changes in the sensorimotor nervous system (Flor et al., 1995, Makin et al., 2013, Mercier and Leonard, 2011, Raffin et al., 2016, Reilly and Sirigu, 2008). Following this, a surge of behavioural therapies that aim to normalise the representation of the phantom hand have been developed in recent years (MacLachlan et al., 2004, Moseley, 2006, Moseley and Flor, 2012, Ramachandran and Rogers-Ramachandran, 1996). The overarching objective of these behavioural therapies is to relieve PLP by improving the ability to move the phantom limb [e.g., mirror therapy (Chan et al., 2007, Rothgangel Stefan et al., 2011) and graded motor imagery (Moseley, 2006, Thieme et al., 2016)]. The assumption behind these therapies is that increased motor control (or motor imagery) over the phantom hand would cause PLP relief. Despite the large number of PLP therapies relying on this notion, the link between PLP and phantom hand motor control is only recently starting to be uncovered behaviourally (Gagné et al., 2009, Raffin et al., 2012a), or using neuroimaging (Makin et al., 2013, Yanagisawa et al., 2016). Systematic evidence for the role of phantom hand motor control in predicting (let alone modulating) PLP is lacking.

The current study aimed at characterising the assumed link between PLP and phantom hand motor control in fourteen upper-limb amputees suffering from chronic PLP. Functional magnetic resonance imaging (fMRI) was used to further examine the neural correlates of deteriorated phantom hand motor control. Specifically, we investigated the relationship between deteriorated motor control and the representation of the phantom hand in primary sensorimotor cortex.

## 2 Materials and methods

### 2.1 Participants

Fifteen unilateral upper-limb amputees who experienced PLP episodes more than once a week in the month preceding recruitment (mean age ± s.e.m. = 47 ± 3, mean years since amputation ± s.e.m. = 16 ± 3, 6 right arm amputees, 4 females; see Table 1 for demographic and clinical details) and fifteen age- and sex-matched controls (2-handers, age = 46 ± 3, 7 with a dominant left hand, 4 females) were recruited through the Oxford Centre for Enablement and Opcare. In this study, we specifically targeted amputees suffering from relatively high chronic PLP. As such, the variance and range of chronic PLP sampled was reduced in the current study (variance: 670, range: 82) compared to our previous study that demonstrated a relationship between chronic PLP and primary sensorimotor phantom hand representation (variance: 754, range: 94; Kikkert, Johansen-Berg, Tracey, & Makin, 2017). However, we note that this difference in chronic PLP variance was not significant, as assessed using Levene's Test of Equality of Variances [*F*_(1,29)_ = .03, *p* = .86]. Ethical approval was granted by the NHS National Research Ethics service (10/H0707/29) and written informed consent was obtained from all participants prior to the study. Data from one amputee was discarded due to inability to perform the motor task with the phantom hand.

**Table 1:** Demographic and clinical details.

	Age	Age at amp.	Amp. Level	Side/dominant	Chronic PLS	Chronic PLP	Chronic Stump pain	Cause of Amp.	Pros. Usage
A01	43	26	2	R/R	90	70	0	Trauma	5
A02	68	53	2	R/R	25	42.5	0	Trauma	5
A03	36	31	2	R/L	20	40	80	Trauma	0
A04	54	54	2	L/R	90	10	20	Vascular D	3
A05	28	24	1	L/R	15	26.7	5	Trauma	3
A06	52	28	4	L/R	80	35	10	Trauma	5
A07	49	45	2	L/L	80	70	10	Tumour	3
A08	47	17	2	L/R	100	15	3.3	Trauma	2
A09	48	27	2	R/R	100	45	0	Trauma	0
A10	23	18	4	R/R	90	25	0	Trauma	0
A11	49	19	2	L/R	70	50	0	Trauma	5
A12	60	31	2	L/R	70	12.5	0	Trauma	0
A13	56	20	5	L/L	70	70	0	Trauma	5
A14	40	27	2	R/L	100	80	26.7	Trauma	2

Amp. = amputation; Amp. Levels: 1 = shoulder, 2 = above elbow, 3 = through elbow, 4 = below elbow, 5 = wrist and below; Side = side of amputation; Dominant = hand dominance prior to amputation (based on self-report); L = left; R = right; PLS = phantom limb sensation; PLP = phantom limb pain; Vascular D = Vascular disease; Pros. Usage = prosthetics usage: 0 = never, 1 = rarely, 2 = occasionally, 3 = daily (less than 4 hours a day), 4 = daily (more than 4 hours a day), 5 = daily (over 8 hours a day).

Amputees participated in four consecutive testing sessions that were separated by at least one week, as part of a larger study (see https://osf.io/4a5zg/ for full protocol). Here, only methods related to results reported in the current paper are detailed. One amputee completed only three testing sessions. Control participants took part in a single session. To compare between the amputees and controls, the phantom hand was matched to the non-dominant hand of controls, and the intact hand was matched to the dominant hand of controls.

### 2.2 Pain ratings

At the start of the first testing session, amputees rated the frequency of PLP, as experienced within the last year, as well as the intensity of worst PLP experienced during the last week (or in a typical week involving PLP). Chronic PLP was calculated by dividing worst PLP intensity (scale 0–100: ranging from no pain to worst pain imaginable) by PLP frequency (1 – all the time, 2 – daily, 3 – weekly, 4 – several times per month, and 5 – once or less per month). This approach reflects the chronic aspect of PLP as it combines both frequency and intensity (Makin et al., 2015a, Makin et al., 2013, Makin et al., 2015b; see Appendix A: Supplementary materials for further details on this measure's consistency over years). A similar measure was obtained for non-painful phantom sensation vividness and stump pain. Ratings of transient PLP intensity (scale 0–100, as above) were obtained in each testing session prior to the finger-tapping test.

### 2.3 Finger-tapping test

Motor control was assessed using the ‘finger-to-thumb opposition task’ (hereafter finger-tapping task). In this task, participants sequentially opposed each of the four fingertips to the tip of their thumb, starting with the index finger. Participants were instructed to repeat this movement cycle five times, and verbally indicated the ending of each cycle. Participants first performed the finger-tapping task with their intact hand and then repeated the task using their phantom hand. Importantly, phantom hand movements are distinguishable from imagined movements, as is supported by empirical evidence demonstrating that phantom limb movements elicit both central and peripheral motor signals that are different from those found during movement imagery (Makin et al., 2013, Raffin et al., 2012b, Reilly et al., 2006, Raffin et al., 2012a). As such, emphasis was given to making “actual” instead of imagined phantom hand movements. Participants were encouraged to perform the finger-tapping task as well as possible, given their volitional motor control over the fingers. If it was impossible to make the full finger-to-thumb movements with the phantom fingers, participants were asked to attempt to perform the instructed movement. During the task, participants were requested to keep their eyes closed, their intact hand relaxed in their lap and all other body parts still. Note that this task has no spatial components (e.g., Makin et al., 2010, Wilf et al., 2013), and therefore the intact hand position was not expected to modulate task performance.

Participants were further asked to perform the finger-tapping task bimanually, where they used their intact hand to mirror the precise degree and speed of movement of the phantom hand. Lastly, participants were asked to perform the finger-tapping task using imagined intact and phantom hands movements separately.

Response timing for completing the five movement cycles was recorded in real time by an experimenter using a stopwatch, based on participants' verbal reports. To establish a normalised measure for phantom hand movement response time (hereafter phantom hand movement) accounting for inter-subject response variability, the intact hand movement response time was extracted from the phantom hand movement response time.

Upon completion of each trial, participants were asked to rate the movement difficulty (scale 0–100: ranging from easy to most difficult; see Appendix A: Supplementary materials for related results), as well as whether the movement induced transient PLP (scale 0–100, as above). Performing the phantom hand finger-tapping task increased transient PLP in 38% of all trials, with an average PLP increase of 10 points. Intact hand finger-tapping never induced PLP. The bimanual finger-tapping task elicited PLP in 44% of all trials, with an average PLP increase of 10 points. The imagined phantom hand finger-tapping task increased transient PLP in 13% of all trials, with an average PLP increase of 2 points. Imagined intact hand finger-tapping induced PLP in 3% of all trials, with an average PLP increase of 1 point.

### 2.4 Functional magnetic resonance imaging (fMRI) sensorimotor task

Participants were visually instructed to make simple feet (bilateral toes), lips, intact hand (all fingers flexion and extension), and phantom hand (as the intact hand) movements, in a block-design fashion. Each movement condition was repeated four times in a counterbalanced protocol, alternating 12 sec of movement with 12 sec of rest. The movement pace was instructed at .5 Hz. Participants were clearly instructed to make actual rather than imagined phantom hand movements. If it was impossible to perform full phantom hand movements, participants were asked to attempt to perform the movements. By asking amputees to perform phantom hand movements, we directly targeted otherwise latent phantom hand representation in the primary sensorimotor missing hand cortex (Kikkert et al., 2016). We have previously shown that this task is successful in producing primary sensorimotor cortex activity across a heterogeneous group of upper limb amputees (i.e., in terms of PLP, phantom sensations, level of amputation, etc.; Makin et al., 2013). Instructions were delivered visually using Presentation software (version 16.4). Head motion was minimized using padded cushions.

### 2.5 MRI data acquisition and analysis

MRI data acquisition, preprocessing and analysis followed standard procedures, as detailed in Appendix A: Supplementary materials. Functional images were obtained using a multiband T2*-weighted pulse sequence with an acceleration factor of 6 (Moeller et al., 2010, Xu et al., 2013). This provided the opportunity to acquire data with increased spatial (2 mm^3^) and temporal (TR: 1300 msec) resolution.

Data collected for individuals with an amputated (or for controls non-dominant) right hand was flipped on the mid-sagittal plane before all analyses, such that the hemisphere contralateral to the phantom hand was consistently aligned (Bogdanov et al., 2012, Diers et al., 2010, Foell et al., 2014, Lotze et al., 2001, MacIver et al., 2008, Raffin et al., 2012b). Common pre-processing steps for fMRI data were applied to each individual run, using FSL's Expert Analysis Tool FEAT (v6.00) (Jenkinson et al., 2012, Smith et al., 2004, Woolrich et al., 2009).

First-level (time-series) parameter estimates were computed using a voxel-based general linear model (GLM) based on the double-gamma hemodynamic response function (HRF) and its temporal derivatives. Two main contrasts were specified between different task movement conditions: 1) intact (or dominant) hand versus feet, and 2) phantom (or non-dominant) hand versus feet. To investigate a potential relationship between chronic PLP and activity in the cortical phantom hand area, phantom hand movements were also contrasted with rest.

Hand regions of interest (ROIs) were selected based on the control group's average hand movement activity, as detailed in Appendix A: Supplementary materials. The percent signal change was extracted for all voxels underlying the hand ROIs and then averaged across scans for each amputee.

### 2.6 Statistical analysis

Statistical analysis was carried out using SPSS software (version 21) and Matlab (version 9.1). For each measure, cases more than 3 standard deviations from the mean were replaced with within-participant means. Data were inspected for violations of normality using the Shapiro–Wilk test. If normality was violated, non-parametric statistical tests were utilised. Two-tailed significance testing was applied unless stated otherwise and standard approaches were used for statistical analysis, as mentioned in the results section and detailed in Appendix A: Supplementary materials.

## 3 Results

Here we focus on the normalised measure for phantom hand movements, i.e., phantom minus intact hand response times. To confirm that the results were not driven by intact hand response times, results were also examined for phantom hand response times and intact hand response times separately. These results are summarised in Table A.1. All results reported below were similar to phantom hand response times only, unless stated otherwise. Below, we only report results based on *a priori* hypotheses derived from previous research (Gagné et al., 2009, Kikkert et al., 2017, Makin et al., 2013, Raffin et al., 2012a), as described in the introduction. Specifically, we focus on correlations between chronic PLP, phantom hand movement response times and activity in the primary sensorimotor phantom hand cortex. Secondary control analyses showing null results were not adjusted for multiple comparisons. More exploratory analyses (e.g., relating to difficulty of movements, motor imagery response times and bimanual movements response times) are reported in Appendix A: Supplementary materials.

### 3.1 Inter-session consistency

No significant difference in phantom hand movement response times was found across the four sessions [repeated measures analysis of variance (ANOVA); *F*_(3,33)_ = 1.10, *p* = .36]. Phantom hand movement inter-session consistency was further confirmed using intraclass correlations (ICC). ICC values range from 0 to 1: ICC values <.4 are considered poor, .4 to .59 are fair, .6 to .74 are good, and >.75 suggest excellent inter-session consistency (Fleiss, Levin, & Cho Paik, 2003, p. 800). For phantom hand movements, this measure indicated good inter-session consistency with an ICC value of .64 (two-way random-model, consistency type) and 95% confidence interval (CI) = .37–.86 [*F*_(11,33)_ = 8.05, *p* < .001]. Inter-session consistency was only fair for imagined phantom hand movements (see Appendix A: Supplementary materials for full results). Average response times across sessions were used for further analysis. Good inter-session consistency was found for phantom hand activity in the primary sensorimotor phantom hand cortex [phantom hand movements *vs* rest contrast; ICC = .63, 95% CI = .37–.85, *F*_(12,36)_ = 7.80, *p* < .001].

### 3.2 Intact versus phantom hand movements

**Fig. 1:**
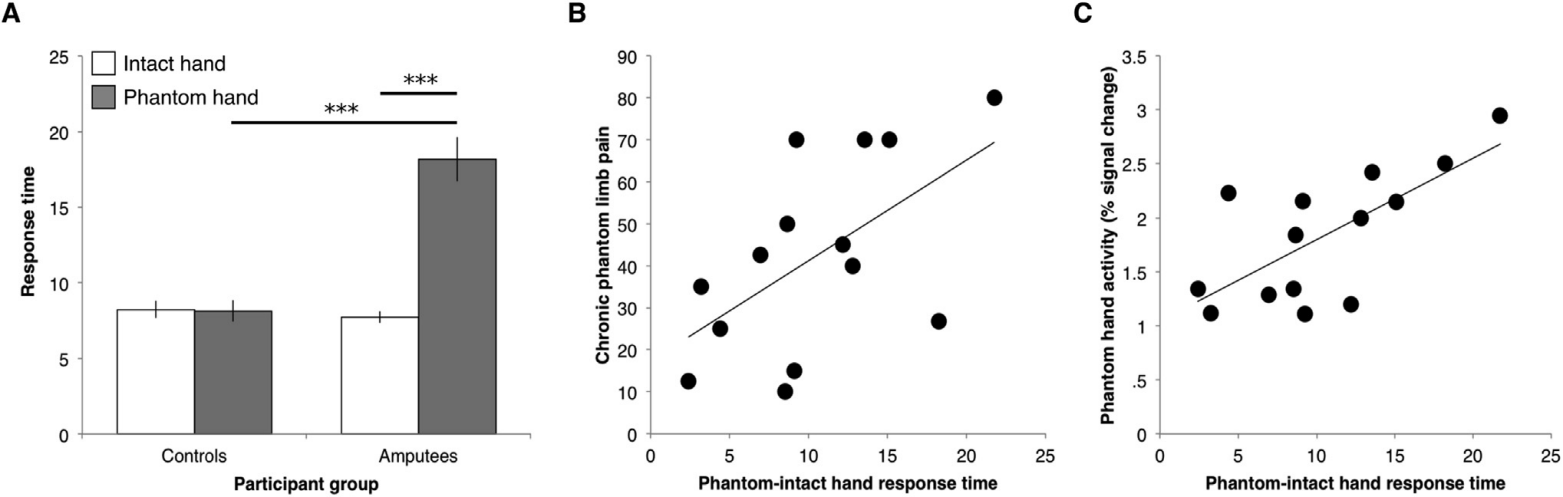
Phantom hand motor control was impaired and related to chronic phantom limb pain and cortical sensorimotor phantom hand representation. (A) Amputees were slower in performing the motor execution task with their phantom hand, both compared to their intact hand and to the non-dominant hand of controls. (B) Amputees experiencing worse chronic PLP took longer to perform the finger-tapping task with their phantom hand (*r* = .57, *p* = .03). (C) Amputees that took longer to perform the finger-tapping task with their phantom hand showed stronger activity in the primary sensorimotor phantom hand cortex when moving their phantom hand (*r* = .66, *p* = .01). Asterisks denote *p* < .001. Response time is shown in seconds. Error bars indicate the s.e.m.

Phantom hand movement response times (i.e. the normalised measure for phantom hand movements: phantom minus intact hand response times) were greater in the amputee group compared to the control group [t_(13.62)_ = −6.99, *p* < .001; note that the degrees of freedom are corrected here as the homogeneity of variances assumption was violated, as indicated by Levene's Test for Equality of Variances]. When considering phantom and intact hand response times separately, motor control over the phantom hand was deteriorated, as demonstrated by increased phantom hand movement response times (Fig. 1A, see Fig. A.1A for similar results for difficulty ratings). Amputees' phantom hand response times were slower both compared to intact hand response times [t_(13)_ = −7.01, *p* < .001] and compared to controls' non-dominant hand response times (*U* = 6, *p* < .001). Intact hand response times were not significantly different between amputees and controls (t_(27)_ = .70, *p* = .49) and no difference in response times was found between dominant and non-dominant hand movements in controls (*Z* = −.71, *p* = .48). These results are consistent with previous reports (Raffin, Giraux, et al., 2012).

### 3.3 Correlations with chronic PLP

Phantom hand movement response times associated with chronic PLP (Fig. 1B, see Fig. A.1B for similar results for difficulty ratings). This result is consistent with previous studies (Gagné et al., 2009, Raffin et al., 2012a). Amputees experiencing worse chronic PLP were slower at performing phantom hand movements (*r* = .57, *p* = .03). The linear regression line denoting the relationship between chronic PLP and phantom hand movement response times in Fig. 1B can be defined by *y* = 2.3962*x* + 17.251. This means that for every 1 sec increase in response times there was a 2.3962 point increase in chronic PLP. As an exploratory test, we also examined the links between phantom hand movement response times and other measurements relating to chronic PLP, such as chronic non-painful phantom limb sensations and transient PLP. We observed that the relationship with phantom hand movement response times did not translate to chronic non-painful phantom sensation experience (*r*_s_ = .08, *p* = .79). Furthermore, no significant correlation was found between transient PLP and phantom hand movement response times in the individual sessions (average *r*_s_ = .30, *p* = .30). The observed correlation between chronic PLP and phantom hand movement response times was not driven by PLP evoked by the task, as shown using a partial correlation including task-evoked PLP as a nuisance regressor (*r*_s_ = .54, *p* = .04). A further exploratory analysis revealed that there was no significant correlation between imagined phantom hand movement response times and chronic PLP (*r* = .22, *p* = .46; see Appendix A: Supplementary materials for full results). These results extend previous findings (Gagné et al., 2009, Raffin et al., 2012a), by showing that the link between phantom hand movement response times and chronic PLP is non-transmutable.

### 3.4 Phantom hand representation

Activity in the primary sensorimotor phantom hand cortex associated with phantom hand movement response times (Fig. 1C). Amputees who were slower in performing the finger-tapping task with the phantom hand outside the scanner activated the primary sensorimotor phantom hand cortex more during flexion and extension of all phantom fingers (*r* = .70, *p* = .005). The linear regression line denoting the relationship between phantom hand movement response times and cortical phantom hand activity in Fig. 1C can be defined by *y* = .0754*x* + 1.0439. This means that for every 1 sec increase in response times there is a .0754% signal increase in phantom hand activity. When regressing out task-evoked PLP using a partial correlation, a strong trend towards a correlation between phantom hand activity in the primary sensorimotor phantom hand cortex and phantom movement response times was observed (*r*_s_ = .51, *p* = .06). Correlations between activity in the primary sensorimotor phantom hand cortex and chronic PLP reached significance in the first and second scanning sessions (one-tailed *r* = .55, *p* = .02 and *r* = .48, *p* = .04, respectively), but not in subsequent scanning sessions (third scanning session: *r* = .18, *p* = .26, fourth scanning session: *r* = .20, *p* = .25; Fig. A.3; see Appendix A: Supplementary Materials for further details).

Note that variations in primary sensorimotor phantom hand cortex activity levels across participants did not result from inter-subject differences in task difficulty: First, phantom hand movements used in the neuroimaging task were customised per participant such that they were comfortable to perform for all participants. Second, the correlation between phantom hand movement response times and cortical sensorimotor activity was independent of difficulty ratings in the finger-tapping task (partial correlation, regressing out difficulty ratings: *r* = .63, *p* = .03). This confirms that the observed increased activity in the primary sensorimotor phantom hand cortex reflected movement representation, and not difficulty.

The correlation between response times and activity in the primary sensorimotor cortex was not significant for the intact hand or for controls (see Appendix A: Supplementary materials for details). Although suggestive, the observed relationship with phantom hand movements might reflect abnormal movement representation, potentially pointing at aberrant processing.

## 4 Discussion

Previous studies reported that chronic PLP positively correlated with the duration of movement execution with the phantom hand (Gagné et al., 2009), as well as difficulty. Furthermore, it was shown that this relationship with chronic PLP did not hold for imagined phantom hand movements (Raffin, Giraux, et al., 2012). In the current study, we confirm and extend these initial findings. First, we validate the reliability of phantom hand movement response times in the finger-tapping task by demonstrating good inter-session consistency. We therefore propose that this measure offers a means to quantify phantom hand motor control. Second, we show that deteriorated phantom hand motor control (i.e., slower response times) positively associated with the strength of cortical sensorimotor phantom hand representation, suggesting that deteriorated phantom hand motor control may be rooted in aberrant cortical representation of the phantom hand. Third, we demonstrate that phantom hand movements are associated with chronic PLP, but not transient PLP or chronic non-painful phantom sensations, thus consolidating the exclusive link between phantom hand motor control and chronic PLP.

Over the past decades various theories have been proposed to explain the neural mechanisms underlying chronic PLP within the context of motor control and sensory inputs. For example, PLP has been suggested to be caused by a incongruency between motor and sensory signals (Harris, 1999, McCabe et al., 2005), problems in the cortical body matrix representation (Moseley, Gallace, & Spence, 2012), a vicious cycle between pain and avoidance behaviour (in this case phantom hand movements; Vlaeyen & Linton, 2000) or prediction errors (Mohan & Vanneste, 2016). We wish to highlight the maintenance of nociceptive peripheral signals following amputation, previously shown to drive PLP (Vaso et al., 2014), as a potential source for the observed association between PLP and deteriorated motor control. It is possible that aberrant inputs from the residual nerves to the primary sensorimotor phantom hand cortex [e.g., through ectopic firing (Nystrom & Hagbarth, 1981)] also disrupt the functioning of the sensorimotor system, leading to deteriorated phantom hand motor control. As such, the current results are in line with our previous neuroimaging findings that link chronic PLP with activity in the primary sensorimotor phantom hand cortex during phantom hand movements (as originally shown in Makin et al. (2013) and replicated in Kikkert et al. (2017)). Here we did not observe a consistent significant correlation between chronic PLP and activity in the cortical phantom area. This could potentially be explained by the restricted range of chronic PLP sampled in the current study, as we specifically targeted individuals with relatively high chronic PLP. When the variation in chronic PLP is reduced, this can explain less variation in brain activity, leading to a lower correlation coefficient. Indeed, lower variability is known to reduce the sensitivity of identifying correlations (Bland & Altman, 2011). As such, further research is needed to determine whether the observed relationship between deteriorated phantom hand motor control and chronic PLP is mediated by the cortical sensorimotor representation of the phantom hand.

The accumulating evidence for a correlation between phantom hand motor control and chronic PLP highlights the importance of studying phantom hand motor control as a feature of chronic PLP, and provides opportunities for refining currently available clinical applications. Current behavioural therapies aiming to relieve PLP through phantom limb movement therapy (e.g., mirror therapy and graded motor imagery) have shown mixed effectiveness (Bowering et al., 2013, Ortiz-Catalan et al., 2016, Thieme et al., 2016; for related results of mirror therapy for complex regional pain syndrome, see Bowering et al., 2013, Moseley et al., 2008). While these therapies are based on the assumption that increased motor control over the phantom hand can cause a change in PLP, many of these therapies make use of motor imagery, rather than motor execution. Despite the mounting evidence linking phantom hand motor execution and PLP, the existence of a link between phantom hand motor imagery and chronic PLP remains tenuous, and our current findings highlight the diminished consistency of motor imagery performance (see Appendix A: Supplementary materials and Raffin, Giraux, et al., 2012). It is therefore possible that phantom limb movement therapy outcomes could be improved when using actual, instead of imagined, phantom movements in rehabilitation approaches. An alternative explanation for the limited effectiveness of phantom limb movement therapies is that the observed link between phantom hand movements and chronic PLP may not be causal. Indeed, insufficient evidence currently exists to support the assumed causality of this link.

The motor test investigated in this study provides an option for implicit, and potentially more objective, measurement of chronic PLP. Since no implicit measure currently exist for assessing chronic PLP, clinicians rely solely on self-report for diagnostics and monitoring of treatment outcomes. Self-report is known to sometimes be unreliable, biased (Paulhus & Vazire, 2005) and influenced by mood states (Berna et al., 2010, Schweinhardt et al., 2008, Wiech and Tracey, 2009). In certain circumstances (e.g., when determining the impact of a novel treatment through longitudinal pain ratings) our motor task may provide an implicit proxy measure that is more resistant to the confounds sometimes inherent to self-report, as has been shown to be useful in several previous studies exploring analgesic efficacy (Iannetti et al., 2005, Tracey, 2013, Wanigasekera et al., 2016). A potential confound of our approach is that performing the phantom hand finger-tapping test increased transient PLP in a subset of the amputees, and one participant was unable to perform the task. For amputees who are unable to move the phantom hand, performing the task using motor imagery could be an alternative (though sub-optimal) option, but more research is needed to validate this approach.
