# Supplementary material for: Motor correlates of phantom limb pain
Source: Cortex. 2017 Oct;95:29–36. doi: 10.1016/j.cortex.2017.07.015 (PMC5637164; doi:10.1016/j.cortex.2017.07.015)
Supplement: Supplementary file 1 [file mmc1.docx]

**Appendix A: Supplementary Materials**

**A.1 Supplementary methods**.

**A.1.1 Pain ratings**

Chronic PLP was calculated by dividing worst PLP intensity (scale 0 - 100: ranging from no pain to worst pain imaginable) by PLP frequency (1 - all the time, 2 - daily, 3 - weekly, 4 - several times per month, and 5 - once or less per month). As we have now used this measure in multiple studies (Kikkert, Johansen-Berg, Tracey, & Makin, n.d.; Makin et al., 2013), we are now able to assess consistency of this measure across studies (i.e. over years). When considering all amputees that participated in at least two of our studies (n = 21), we found excellent inter-studies consistency for our measure of chronic PLP (ICC = 0.88, 95% CI = 0.72-0.95, F_(20,20)_ = 15.42, p < 0.001). We also found excellent inter-studies consistency when considering amputees that participated in at least three of our past studies (n = 9; ICC = 0.78, 95% CI = 0.48-0.94, F_(8,16)_ = 11.67, p < 0.001).

**A.1.2 MRI data acquisition**

MRI images were collected by means of a 3-Tesla Verio MRI scanner (Siemens, Erlangen, Germany), using a 32-channel head coil. A high-resolution T1-weighted sequence was used to acquire a structural image (TR: 2040ms, TE: 4.7ms, flip angle 8°, voxel size: 1mm isotropic resolution). Functional images based on the blood oxygenation level-dependent (BOLD) signal were obtained using a multiband T2*-weighted pulse sequence with an acceleration factor of 6 (Moeller et al., 2010; Xu et al., 2013). The following acquisition parameters were used: TR: 1300ms, TE: 40ms, spatial resolution: 2 mm^3^, flip angle: 66°, 72 transversal slices, 314 acquired volumes. A high-saturation first volume of each acquired multiband run was collected for registration purposes. Field maps were acquired for field unwarping.

**A.1.3 MRI preprocessing and image registration**

Data collected for individuals with an amputated (or non-dominant for controls) right hand was flipped on the mid-sagittal plane before all analyses, such that the hemisphere corresponding to the phantom hand was consistently aligned (Bogdanov, Smith, & Frey, 2012; Diers, Christmann, Koeppe, Ruf, & Flor, 2010; Foell, Bekrater-Bodmann, Diers, & Flor, 2014; Lotze, Flor, Grodd, Larbig, & Birbaumer, 2001; MacIver, Lloyd, Kelly, Roberts, & Nurmikko, 2008; Raffin, Mattout, Reilly, & Giraux, 2012). This procedure is regularly exercised in functional connectivity studies involving lateralised populations (Douaud, Filippini, Knight, Talbot, & Turner, 2011; Park et al., 2011). Common pre-processing steps for fMRI data were applied to each individual run, using FSL’s Expert Analysis Tool FEAT (v6.00; fsl.fmrib.ox.ac.uk/fsl/fslwiki) (Jenkinson, Beckmann, Behrens, Woolrich, & Smith, 2012; Smith et al., 2004; Woolrich et al., 2009): motion correction using FMRIB’s Linear Image Registration Tool (MCFLIRT (Jenkinson, Bannister, Brady, & Smith, 2002)), brain extraction using automated brain extraction tool BET (Smith, 2002), spatial smoothing using a 3mm FWHM (full width at half maximum) Gaussian kernel, and high pass temporal filtering with a cut-off of 100s.

The first volume of each acquired multiband scan was used as an alternative reference image to enhance registration purposes. Functional images were registered to the example functional image of the acquired run in the first imaging session for each participant (7 degrees of freedom). This example functional image was then aligned to the structural image acquired in the first imaging session for each participant, initially using linear registration (FMRIB’s Linear Image Registration Tool, FLIRT (Jenkinson et al., 2002; Jenkinson & Smith, 2001)) and optimised using boundary based registration (Greve & Fischl, 2009). Structural images were transformed to MNI standard space using nonlinear registration tool FNIRT, and the resulting warp fields were applied to the functional statistical images.

Time series statistical analysis was carried out using FILM (FMRIB’s Improved Linear Model) with local autocorrelation correction. Estimated head motion parameters (as estimated by MCFLIRT) were added to the model to remove residual motion effects. All fMRI data was further assessed for excessive motion using motion estimate outputs from MCFLIRT: volumes with estimated absolute mean displacement greater than 1mm (half of the functional voxel size) were scrubbed (amputees: scrubbing applied in 26% of all scans, maximum percentage of volumes scrubbed in a given session: 1.6%; controls: scrubbing applied in 6.7% of all scans, maximum percentage of volumes scrubbed in a given session: 1%).

**A.1.4 MRI region of interest selection**

Correlational analysis was carried out using a region of interest (ROI) approach. To define the two sensorimotor hand region areas, an average spatial map for the control group was created using FMRIB’s Local Analysis of Mixed Effects (FLAME). A mask was drawn around the anatomical hand knob, extending the pre- and postcentral gyrus and the central sulcus. Within this mask, the top 400 voxels of the controls’ average spatial map for each of the two main contrasts (dominant hand versus feet movements; non-dominant hand versus feet movements) were combined to form the two hand ROIs.

**A.1.5 Statistical analysis**

Between groups comparisons were assessed using independent samples two-tailed student *t*-tests. If the homogeneity of variances assumption was violated, as indicated by Levene’s test for equality of variances, the Welch-Satterthwaite method was used to adjust the degrees of freedom. Within groups, means were statistically compared using paired two-tailed student *t*-tests. When significant departure from normality was found (based on the Shapiro-Wilk test), nonparametric tests were used (Man-Whitney U test or Wilcoxon signed rank test, as appropriate). Correlations were evaluated using two-tailed Pearson’s correlation coefficients, or a Spearman rank-order correlation coefficient if normality assumptions were violated. We further calculated two-way random single measures of intraclass correlations (ICCs) to assess consistency between sessions.

**A.2 Supplementary results**

As in the main results section, here we focus on the normalised measure for phantom hand movements, i.e. phantom minus intact hand response times. To confirm that the results were not driven by intact hand response times, results were also examined for phantom hand response times and intact hand response times alone. These results are summarised in Table A.1. All results reported below were similar for phantom hand response times only, unless stated otherwise.

**A.2.1 Bimanual test performance as a measure for task consistency**

Besides showing inter-session consistency by means of the ICC measure, test consistency for phantom hand movements was confirmed by demonstrating a strong correlation in response times between bimanual and phantom hand movements (phantom minus intact hand: r = 0.77, p = 0.001; phantom hand: r = 0.81, p < 0.001). No such correlation was found between bimanual and intact hand movements (r = 0.11, p = 0.71). Amputees were significantly slower in performing the bimanual task, both compared to the phantom and intact hands (t_(13)_ = 6.06, p < 0.001 and t_(13)_ = 3.32, p = 0.005, respectively).

**A.2.2 Movement execution difficulty**

Movement difficulty ratings showed a similar pattern of results as those of the response times. Inter-session consistency for phantom hand movement difficulty was considered fair, as indicated by an ICC value of 0.52 with 95% CI = 0.25-0.79 (F_(12,36)_ = 5.36, p < 0.001) (Fleiss, Levin, & Cho Paik, 2003). Average difficulty ratings were used for further analysis.

Results obtained using difficulty ratings reflected deteriorated phantom hand motor control (Figure A.2). Amputees’ perceived phantom hand movements as more difficult compared to the control group (U = 0, p < 0.001). When considering phantom hand and intact hand movement difficulty separately, amputees’ phantom hand movements were perceived as more difficult, both compared to the intact hand (Z = -3.18, p = 0.001) and compared to controls (U = 24.50, p < 0.001). No difference in difficulty was found between dominant and non-dominant hand movements in controls (Z = -1.14, p = 0.26).

There was a trend towards a relationship between phantom hand movement difficulty and chronic PLP. Amputees experiencing worse chronic PLP found it more difficult to perform phantom hand movements (r = 0.50, p = 0.08). No relationship existed between phantom hand movement difficulty and chronic phantom sensation experience (r_s_ = -0.39, p = 0.19). Furthermore, no significant correlation was found between phantom hand movement difficulty in the individual sessions and transient PLP (average r_s_ = 0.07, p = 0.81).

**A.2.3 Movement imagery as a surrogate measure for phantom limb pain**

Overall, the movement imagery task resulted in a similar, though less robust, pattern of results as the movement execution task. No significant difference in phantom hand movement imagery response times was found across the four sessions (repeated measures ANOVA; F_(3,33)_ = 0.20, p = 0.90). The ICC measure revealed fair inter-session consistency for phantom hand movement imagery response times (ICC = 0.45 with 95% CI = 0.16-9.75, F_(11,33)_ = 4.2, p = 0.001) (Fleiss et al., 2003). Average response times across sessions were used for further analysis.

Phantom hand movement imagery and phantom hand movement execution response time were correlated (phantom minus intact hand: r = 0.58, p = 0.03; phantom hand: r = 0.51, p = 0.06). This was not true for the intact hand (r = 0.40, p = 0.16).

Results from the movement imagery task were dissimilar to those of the movement execution task (Figure A.2A). No difference in response time was found between amputees and controls for phantom hand movement imagery (i.e. phantom minus intact hand; U = 73, p = 0.16). Amputees took longer to perform the movement imagery task compared to controls, both with their phantom and intact hand (U = 32, p = 0.001 and U = 52, p = 0.02, respectively). While controls were equally fast with the two hands in performing the movement imagery task (Z = -1.36, p = 0.17), amputees were slower when performing the movement imagery task with their phantom hand compared to their intact hand (t_(13)_= 2.59, p = 0.02).

No relationship was found between the normalised measure for phantom hand movement imagery response time and chronic PLP (Figure A.2B; r = 0.22, p = 0.46). A trend towards a relationship with chronic PLP was however found when considering phantom hand movement imagery response time separately (r = 0.52, p = 0.06). There was no significant correlation between phantom hand movement imagery and chronic phantom sensation (r_s_ = 0.10, p = 0.73). Furthermore, there was no correlation between the normalised measure for phantom hand movement imagery and transient PLP in the individual sessions (average r_s_= 0.12, p = 0.68). A trend towards a relationship with transient PLP was however found when considering phantom hand movement imagery response times separately (average r_s_ = 0.52, p = 0.06). This is consistent with previous findings, showing a positive, though non-significant relationship between phantom hand movement imagery and chronic PLP(Raffin, Giraux, & Reilly, 2012).

**A.2.4 Chronic phantom limb pain may positively scale with activity in the cortical phantom hand area**

A positive correlation was found between chronic PLP and activity in the cortical phantom hand area in the first and second sessions (one-tailed r = 0.55, p = 0.02 and r = 0.48, p = 0.04 respectively). Amputees experiencing worse chronic PLP showed stronger activity in the cortical phantom hand area during flexion and extension of all phantom fingers (Figure A.3 for results of the first session). No significant relationship with chronic PLP was found for the third and fourth scanning sessions (one-tailed r = 0.18, p = 0.26 and r = 0.20, p = 0.25 respectively), or for the averaged cortical phantom hand activity (across sessions; r = 0.30, p = 0.28).

**A.2.5 Representation of controls’ hands and amputees’ intact hand**

No correlation was found between intact hand response time in the finger-tapping task and activity in the cortical sensorimotor intact hand area during flexion and extension of all fingers of the intact hand (r = 0.36, p = 0.20). Furthermore, no relationship was found between the cortical sensorimotor hand representations and finger-tapping response times in controls (non-dominant minus dominant hand response time & non-dominant hand representation: r = -0.27, p = 0.32; non-dominant hand response time & non-dominant hand representation: r_s_ = 0.20, p = 0.48; dominant hand response time & dominant hand representation: r = -0.27, p = 0.34).

**Supplementary references**

Bogdanov, S., Smith, J., & Frey, S. H. (2012). Former Hand Territory Activity Increases After Amputation During Intact Hand Movements, but Is Unaffected by Illusory Visual Feedback. *Neurorehabilitation and Neural Repair*, *26*(6), 604–615. http://doi.org/10.1177/1545968311429687

Diers, M., Christmann, C., Koeppe, C., Ruf, M., & Flor, H. (2010). Mirrored, imagined and executed movements differentially activate sensorimotor cortex in amputees with and without phantom limb pain. *Pain*, *149*(2), 296–304. http://doi.org/10.1016/j.pain.2010.02.020

Douaud, G., Filippini, N., Knight, S., Talbot, K., & Turner, M. R. (2011). Integration of structural and functional magnetic resonance imaging in amyotrophic lateral sclerosis. *Brain : A Journal of Neurology*, *134*(Pt 12), 3470–9. http://doi.org/10.1093/brain/awr279

Fleiss, J., Levin, B., & Cho Paik, M. (2003). Statistical Methods for Rates and Proportions. *John Wiley & Sons*, 800. http://doi.org/10.1198/tech.2004.s812

Foell, J., Bekrater-Bodmann, R., Diers, M., & Flor, H. (2014). Mirror therapy for phantom limb pain: brain changes and the role of body representation. *European Journal of Pain (London, England)*, *18*(5), 729–739. http://doi.org/10.1002/j.1532-2149.2013.00433.x

Greve, D. N., & Fischl, B. (2009). Accurate and robust brain image alignment using boundary-based registration. *NeuroImage*, *48*(1), 63–72. http://doi.org/10.1016/j.neuroimage.2009.06.060

Jenkinson, M., Bannister, P., Brady, M., & Smith, S. M. (2002). Improved optimization for the robust and accurate linear registration and motion correction of brain images. *NeuroImage*, *17*(2), 825–841. http://doi.org/10.1016/S1053-8119(02)91132-8

Jenkinson, M., Beckmann, C. F., Behrens, T. E. J., Woolrich, M. W., & Smith, S. M. (2012). FSL. *NeuroImage*, *62*(2), 782–790. http://doi.org/10.1016/j.neuroimage.2011.09.015

Jenkinson, M., & Smith, S. M. (2001). A global optimization method for robust affine registration of brain images. *Medical Imaging Analysis*, *5*, 143–156.

Kikkert, S., Johansen-Berg, H., Tracey, I., & Makin, T. R. (n.d.). Reaffirming the link between chronic phantom limb pain and maintained missing hand representation. *Submitted*.

Lotze, M., Flor, H., Grodd, W., Larbig, W., & Birbaumer, N. (2001). Phantom movements and pain. An fMRI study in upper limb amputees. *Brain*, *124*(Pt 11), 2268–2277.

MacIver, K., Lloyd, D. M., Kelly, S., Roberts, N., & Nurmikko, T. (2008). Phantom limb pain, cortical reorganization and the therapeutic effect of mental imagery. *Brain : A Journal of Neurology*, *131*(Pt 8), 2181–91. http://doi.org/10.1093/brain/awn124

Makin, T. R., Filippini, N., Duff, E. P., Henderson Slater, D., Tracey, I., & Johansen-Berg, H. (2015). Network-level reorganisation of functional connectivity following arm amputation. *NeuroImage*, *114*, 217–225. http://doi.org/10.1016/j.neuroimage.2015.02.067

Makin, T. R., Scholz, J., Filippini, N., Henderson Slater, D., Tracey, I., & Johansen-Berg, H. (2013). Phantom pain is associated with preserved structure and function in the former hand area. *Nature Communications*, *4*, 1570. http://doi.org/10.1038/ncomms2571

Makin, T. R., Scholz, J., Henderson Slater, D., Johansen-Berg, H., & Tracey, I. (2015). Reassessing cortical reorganization in the primary sensorimotor cortex following arm amputation. *Brain*, *138*(8), 2140–2146. http://doi.org/10.1093/brain/awv161

Moeller, S., Yacoub, E., Olman, C. A., Auerbach, E., Strupp, J., Harel, N., & Ugurbil, K. (2010). Multiband multislice GE-EPI at 7 tesla, with 16-fold acceleration using partial parallel imaging with application to high spatial and temporal whole-brain FMRI. *Magnetic Resonance in Medicine*, *63*(5), 1144–1153. http://doi.org/10.1002/mrm.22361

Park, C., Chang, W. H., Ohn, S. H., Kim, S. T., Bang, O. Y., Pascual-Leone, A., & Kim, Y.-H. (2011). Longitudinal changes of resting-state functional connectivity during motor recovery after stroke. *Stroke; a Journal of Cerebral Circulation*, *42*(5), 1357–62. http://doi.org/10.1161/STROKEAHA.110.596155

Raffin, E., Giraux, P., & Reilly, K. T. (2012). The moving phantom: Motor execution or motor imagery? *Cortex*, *48*(6), 746–757. http://doi.org/10.1016/j.cortex.2011.02.003

Raffin, E., Mattout, J., Reilly, K. T., & Giraux, P. (2012). Disentangling motor execution from motor imagery with the phantom limb. *Brain*, *135*(2), 582–595. http://doi.org/10.1093/brain/awr337

Smith, S. M., Jenkinson, M., Woolrich, M. W., Beckmann, C. F., Behrens, T. E. J., Johansen-Berg, H., … Matthews, P. M. (2004). Advances in functional and structural MR image analysis and implementation as FSL. *NeuroImage*, *23*(SUPPL. 1), 208–219. http://doi.org/10.1016/j.neuroimage.2004.07.051

Woolrich, M. W., Jbabdi, S., Patenaude, B., Chappell, M., Makni, S., Behrens, T. E. J., … Smith, S. M. (2009). Bayesian analysis of neuroimaging data in FSL. *NeuroImage*, *45*(1 Suppl), S173–S186. http://doi.org/10.1016/j.neuroimage.2008.10.055

Xu, J., Moeller, S., Auerbach, E. J., Strupp, J., Smith, S. M., Feinberg, D. A., … Uǧurbil, K. (2013). Evaluation of slice accelerations using multiband echo planar imaging at 3T. *NeuroImage*, *83*, 991–1001. http://doi.org/10.1016/j.neuroimage.2013.07.055

**Supplementary Figures & Tables**

***
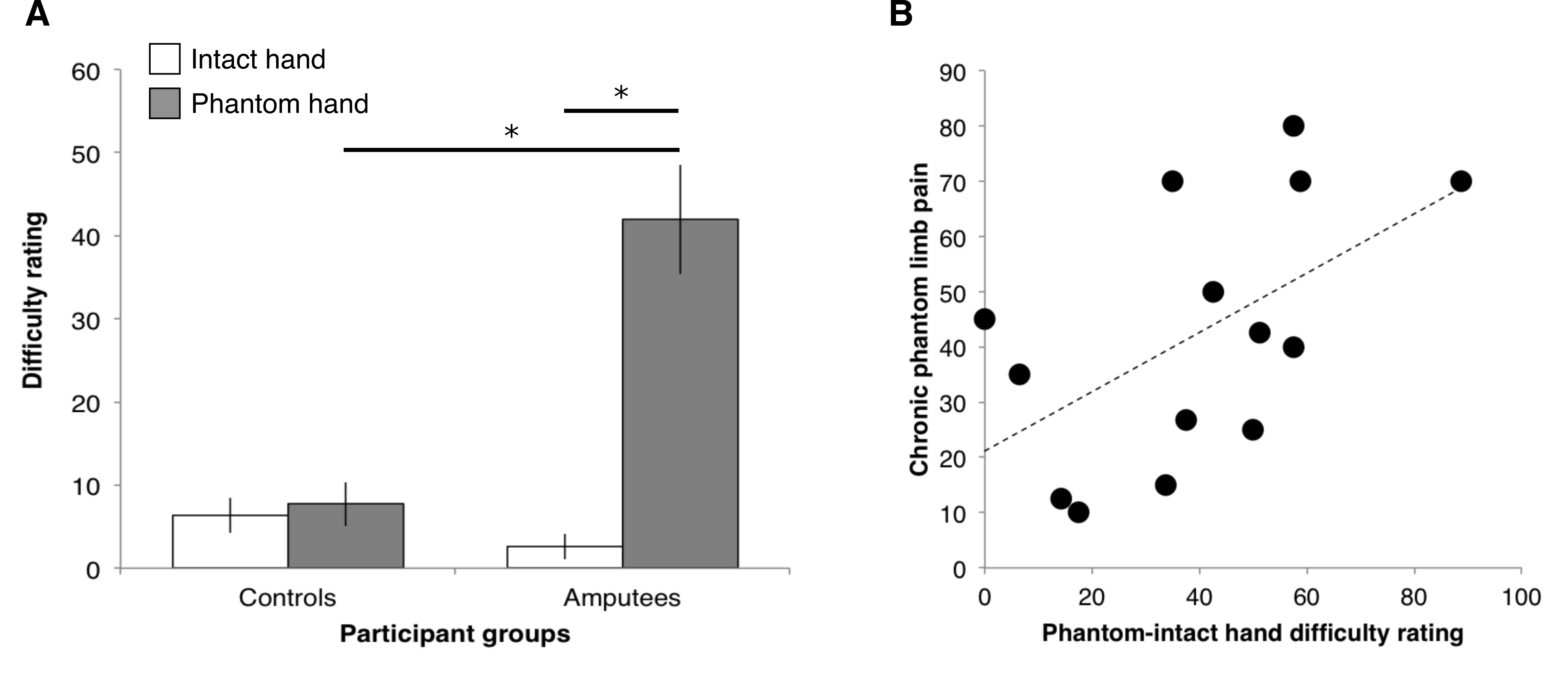
Figure A.1: Phantom hand movement difficulty is deteriorated and relates to chronic phantom limb pain.*** (A) Amputees found it more difficulty to perform the task with their phantom hand, both compared to their intact hand and to movement difficulty of the non-dominant hand of controls. (B) There was a trend towards a relationship between phantom hand movement difficulty and chronic phantom limb pain (r = 0.50, p = 0.08). Asterisks denote p < 0.001. Response time is shown in seconds. Error bars indicate the s.e.m.

***
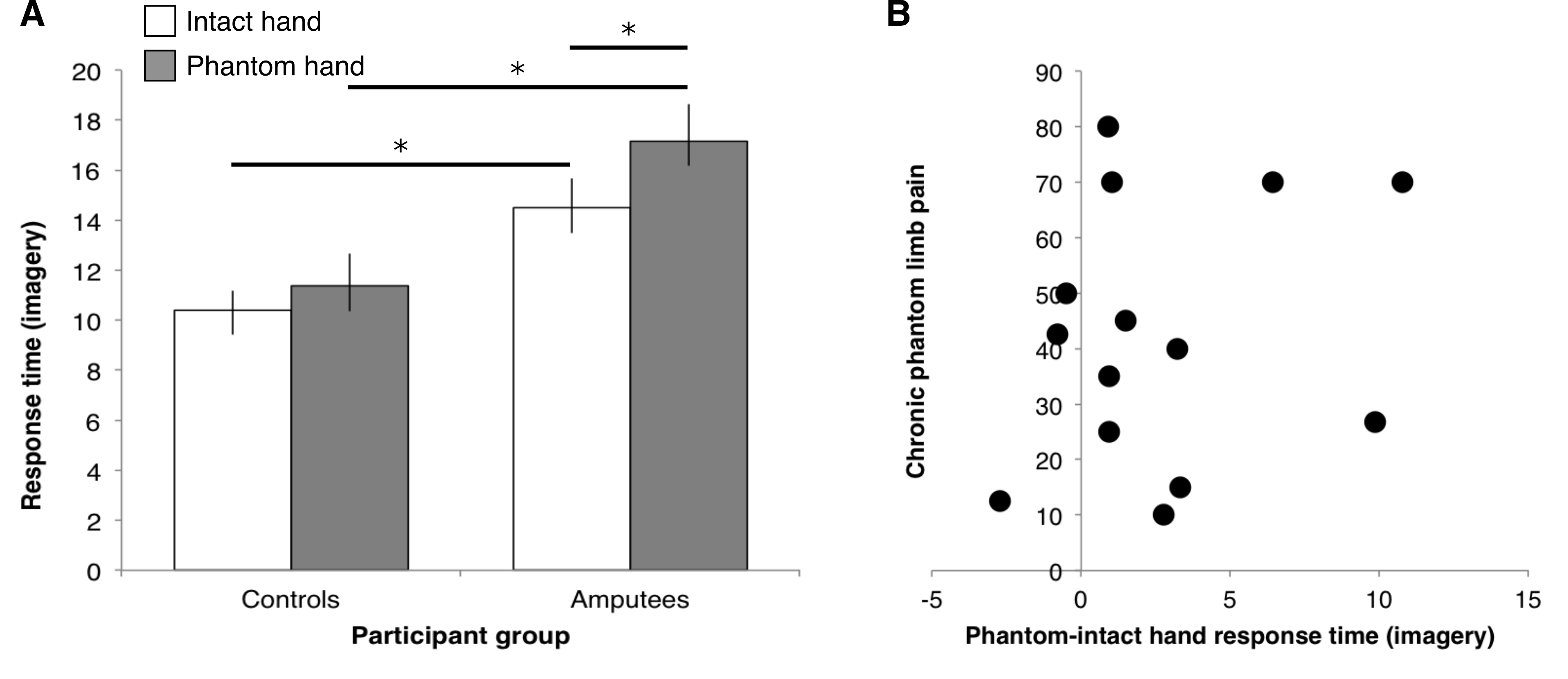
Figure A.2: Phantom hand motor imagery is altered in amputees, but this does not correlate with chronic phantom limb pain.*** (A) Amputees were slower at performing the imagined finger-tapping task, compared to controls. It took amputees longer to perform motor imagery with their phantom hand, compared to their intact hand. (B) There was no significant relationship between phantom hand motor imagery and chronic phantom limb pain (r_s_= 0.12, p = 0.68). Asterisks denote p < 0.05. Response time is shown in seconds. Error bars indicate the s.e.m.

***
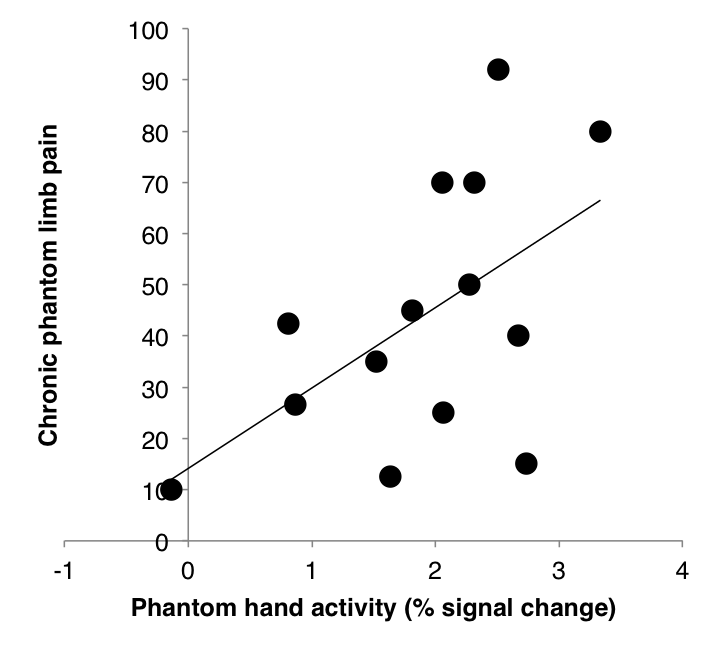
***

***Figure A.3: Chronic phantom limb pain associates with the cortical sensorimotor phantom hand representation.*** There was a significant relationship between chronic phantom limb pain and activity in the cortical phantom hand area in the first session (r = 0.55, p = 0.02). Amputees who activated the cortical phantom hand area more during flexion and extension of all phantom fingers experienced worse chronic phantom limb pain.

|  | Inter-session relationships | | | Correlations with phantom limb phenomena | | | Correlations with activity in cortical sensorimotor hand area | |
| --- | --- | --- | --- | --- | --- | --- | --- | --- |
|  | **Average Pearson** | **ICC** | | **Chronic PLP** | **Chronic non-painful PLS** | **Transient PLP** | **Phantom hand area** | **Intact hand area** |
|  |  | **ICC-value** | **ICC-CI** |  |  |  |  |  |
| Response times  Execution |  |  |  |  |  |  |  |  |
| *Phantom-intact hand* | 0.68** | 0.64 | 0.37-0.86 | 0.57* | ^a^0.08 | ^a^0.30 | 0.70** | - |
| *Phantom hand* | 0.62* | 0.60 | 0.33-0.84 | 0.62* | ^a^0.06 | ^a^0.38 | 0.61* | - |
| *Intact hand* | 0.78** | 0.80 | 0.60-0.93 | 0.15 | - | - | - | 0.36 |
| Imagination |  |  |  |  |  |  |  |  |
| *Phantom-intact hand* | ^a^0.35 | 0.45 | 0.16-0.75 | 0.22 | ^a^0.10 | ^a^0.12 | - | - |
| *Phantom hand* | 0.60* | 0.58 | 0.30-0.83 | 0.52 | ^a^-0.10 | ^a^0.52 | - | - |
| *Intact hand* | 0.70** | 0.67 | 0.41-0.87 | 0.45 | - | - | - | - |
| Difficulty ratings  Execution |  |  |  |  |  |  |  |  |
| *Phantom-intact hand* | 0.55* | 0.52 | 0.25-0.79 | 0.50 | ^a^-0.39 | ^a^0.07 | - | - |
| *Phantom hand* | ^a^0.55* | 0.54 | 0.27-0.80 | 0.49 | ^a^-0.45 | ^a^0.06 | - | - |
| *Intact hand* | ^a^0.70** | 0.79 | 0.60-0.92 | ^a^-0.42 | - | - | - | - |

**Table A.1: Statistics for phantom and intact hand measures separately.** Intraclass correlation (ICC) values range from 0 to 1: we designate ICC values <0.4 as poor, 0.4 to 0.59 as fair, 0.6 to 0.74 as good, and >0.75 as excellent (Fleiss et al., 2003). ICC values designated as poor or fair are printed in red. ICC values designed as good or excellent are printed in green. CI = 95% confidence interval. PLP = phantom limb pain. PLS = phantom limb sensation. Asterisks indicate significance, with: * = p <0.05, ** = p < 0.01, *** = p < 0.001. ^a^ = normality assumption was violated, non-parametric equivalent was used (Spearman).
